# Correlates of psychopathic personality traits in everyday life: results from a large community survey

**DOI:** 10.3389/fpsyg.2014.00740

**Published:** 2014-07-22

**Authors:** Scott O. Lilienfeld, Robert D. Latzman, Ashley L. Watts, Sarah F. Smith, Kevin Dutton

**Affiliations:** ^1^Department of Psychology, Emory UniversityAtlanta, GA, USA; ^2^Department of Psychology, Georgia State UniversityAtlanta, GA, USA; ^3^Department of Experimental Psychology, University of OxfordOxford, UK

**Keywords:** psychopathy, personality, leadership, risk-taking, fearlessness

## Abstract

Although the traits of psychopathic personality (psychopathy) have received extensive attention from researchers in forensic psychology, psychopathology, and personality psychology, the relations of these traits to aspects of everyday functioning are poorly understood. Using a large internet survey of members of the general population (*N* = 3388), we examined the association between psychopathic traits, as measured by a brief but well-validated self-report measure, and occupational choice, political orientation, religious affiliation, and geographical residence. Psychopathic traits, especially those linked to fearless dominance, were positively and moderately associated with holding leadership and management positions, as well as high-risk occupations. In addition, psychopathic traits were positively associated with political conservatism, lack of belief in God, and living in Europe as opposed to the United States, although the magnitudes of these statistical effects were generally small in magnitude. Our findings offer preliminary evidence that psychopathic personality traits display meaningful response penetration into daily functioning, and raise provocative questions for future research.

## Introduction

“There walk among us men and women who are in but not of our world” (p. 101), wrote psychiatrist Lindner (1956) over a half century ago. Lindner was describing people with the personality traits of psychopathy (psychopathic personality), who have since become a prime focus of research and clinical attention (see Patrick, 2006; Salekin and Lynam, 2011; Skeem et al., 2011, for reviews). As delineated in the classic clinical descriptions of Cleckley (1941) and Karpman (1941) and elaborated in the writings of Hare (1970, 1993) and Lykken (1995), psychopaths are hybrid creatures. They are superficially charming and commonly make positive first impressions on others. At the same time, they are callous, grandiose, guiltless, and dishonest, and frequently engage in impulsive and reckless acts (McCord and McCord, 1964). Their romantic relationships are typically devoid of tender emotions and marked by rampant promiscuity (Hare, 1993).

### Psychopathy in community samples

Data from taxometric studies suggest that psychopathic traits fall along a dimension and differ from normality in degree rather than kind (Edens et al., 2006), bolstering the argument for examining these traits in community samples (e.g., Neumann and Hare, 2008). Indeed, there is burgeoning evidence that psychopathic traits, such as impulse control deficits and fearlessness, display many of the same correlates in community samples as in prison samples (e.g., Benning et al., 2005).

Despite the growing interest in investigating the correlates of psychopathic traits in non-criminal samples (Hall and Benning, 2006; see also Widom, 1977), surprisingly little is known about the implications of these traits for everyday functioning, such as occupational choice, leadership positions, political orientation, religious belief, or place of residence. In this article, we aim to make preliminary inroads into these largely unaddressed questions. Given that psychopathic personality traits are associated with a host of maladaptive correlates, such as physical aggression (Leistico et al., 2008) and substance misuse (Smith and Newman, 1990), and perhaps certain adaptive correlates, such as successful leadership (Lilienfeld et al., 2012b) and perseverance (Latzman et al., in press), the extent to which such traits display response penetration (see Tellegen, 1991) in daily life is of theoretical and pragmatic importance.

### Self-reported psychopathy in community samples

Although the most widely used and arguably best validated measure of psychopathy is the Psychopathy Checklist-Revised (PCL-R; Hare, 1991/2003), a semi-structured interview that incorporates corroborative information, this measure is typically appropriate only for prison and jail samples in which file data are readily available. The increasing interest in examining psychopathy in non-clinical samples has coincided with the development of several well-validated self-report measures of this condition suitable for use outside of prison walls (e.g., Levenson et al., 1995; see Lilienfeld and Fowler, 2006, for a review). Nevertheless, we focus on one such measure here, namely, the Psychopathic Personality Inventory (PPI; Lilienfeld and Andrews, 1996), now the Psychopathic Personality Inventory-Revised (PPI-R; Lilienfeld and Widows, 2005). The PPI and PPI-R, which are arguably the most widely used questionnaire measures of psychopathy, contain eight lower-order subscales designed to detect specific personality features of psychopathy. The PPI and PPI-R were constructed explicitly for application to non-clinical and non-criminal samples. They focus on the core affective and interpersonal traits of psychopathy (e.g., callousness, guiltlessness, fearlessness, superficial charm, grandiosity) and place minimal evidence on overt antisocial and criminal behaviors, which are less prevalent in such samples.

Although virtually all early work on psychopathy implicitly regarded the condition as unifactorial and therefore relied on total psychopathy scores (see Hare, 1970 and Hare and Schalling, 1978, for summaries), more recent research has typically identified two broad dimensions underpinning scores on widely used psychopathy measures (Harpur et al., 1989). Specifically, many factor-analytic solutions have identified one factor reflecting the core affective and interpersonal features of psychopathy. Nevertheless, the nature of this factor often differs substantially across psychopathy measures (e.g., Malterer et al., 2010), with the first factor of some measures, such as the PCL-R and its derivatives, primarily assessing callousness with a smaller contribution from boldness, and the first factor of other measures, such as the PPI-R, primarily assessing boldness (Sellbom and Phillips, 2013). Factor analyses have also typically identified a second factor reflecting an antisocial and impulsive lifestyle, or at least a dispositional predisposition toward this lifestyle (e.g., Hare, 1991/2003). Factor analyses of the PPI-R in community samples, for example, have often revealed a higher-order dimension termed Fearless Dominance, which comprises three lower-order subscales that assess physical and social boldness and immunity to anxiety, and another higher-order dimension termed Self-Centered Impulsivity, which comprises four lower-order subscales that assess a narcissistic and reckless tendency to exploit and blame others (Benning et al., 2003; but see Neumann et al., 2008, for an alternative factor structure). Fearless Dominance, which is similar to boldness (Patrick et al., 2009) appears to be associated largely with adaptive functioning, whereas Self-Centered impulsivity, which is similar to disinhibition (Patrick et al., 2009), appears to be associated largely with maladaptive functioning (Lilienfeld et al., 2012a; but see Miller and Lynam, 2012, for a critique). An eighth PPI-R subscale, Coldheartedness, does not load highly on either higher-order dimension and is sometimes examined as a third standalone factor in analyses (Lilienfeld and Widows, 2005). This subscale, which overlaps with the construct of meanness (Patrick et al., 2009), assesses a deficiency in the experience of social emotions, such as empathy, guilt, love, and loyalty.

### Psychopathy and everyday functioning

As noted earlier, little is known about the implications of psychopathic personality traits for occupational choice. In a study of students at a Canadian university, Clow and Scott (2007) reported that criminal justice majors (*n* = 107) obtained significantly higher PPI total scores than did nursing majors (*n* = 67); the differences were most pronounced for the PPI subscale of Machiavellian Egocentricity, a marker of the Self-Centered Impulsivity higher-order dimension. Although these results suggest that students with interests in criminality may be somewhat prone to psychopathy, the authors' focus on only two majors renders this conclusion tentative, especially because nursing students appear to be characterized by low levels of antisocial behavior and perhaps psychopathy (Gough, 1994, p. 679; see also Wilson and McCarthy, 2011, for evidence that business/commerce students display higher psychopathy scores than do students in other majors). Although some authors have conjectured that individuals with psychopathic traits are attracted to occupations characterized by physical risk, such as law enforcement, firefighting, and military combat (e.g., Lykken, 1995; Fowles and Dindo, 2006; Dutton, 2012a), virtually no researchers have investigated this possibility systematically. In the only study to our knowledge to examine this issue, Falkenbach and Tsoukalas (2011) compared the psychopathic personality characteristics of a group of individuals engaged in high-risk, prosocial occupations, such as police officers, emergency personnel, and detectives, with those of incarcerated offenders. They found that individuals in the former group scored higher on both PPI Fearless Dominance and PPI Coldheartedness than did offenders, raising the possibility that physical and social boldness, along with an adaptive capacity to distance oneself emotionally from others in distress, may draw individuals toward law enforcement. Nevertheless, the absence of a normative comparison group renders these results challenging to interpret.

Recently, several investigators have become interested in the relation of psychopathic personality traits to leadership given that at least some of the former traits, such as boldness and social risk-taking, may predispose individuals to seek out and obtain positions of authority (see Smith and Lilienfeld, 2012, for a review). Most research suggests that psychopathic traits are positively associated with workplace deviance (O'Boyle et al., 2012) and destructive leadership and management behavior (Boddy, 2011). Nevertheless, in a psychohistorical analysis of the 42 U.S. presidents up to and including George W. Bush, Lilienfeld et al. (2012b) reported that estimated scores on PPI Fearless Dominance (extracted using multiple regression equations from personality trait data completed by biographical experts on each president) were positively associated with several dimensions of presidential success, including overall leadership, communication ability, and crisis management. In addition, in a study of 203 corporate personnel, Babiak et al. (2010) found that PCL-R scores were associated with both (a) ratings of ineffective management skills and being a poor team player and (b) successful communication and creativity, raising the intriguing possibility that psychopathy is a double-edged sword in the leadership domain. In addition, they mentioned that among high PCL-R scorers, there appeared to be an overrepresentation of individuals in leadership (e.g., company vice-presidents) and management positions, although the numbers were too small to allow inferential statistical comparisons. To our knowledge, there are no other data addressing the question of whether people with high levels of psychopathic traits are more likely than those with low levels to occupy leadership positions. Nevertheless, given that the boldness that some authors regard as important to psychopathy (Lilienfeld et al., 2012a; cf., Lynam and Miller, 2012) may bear important implications for leadership and management positions, this possibility is worthy of investigation.

In addition, no published data appear to be available concerning the relation of psychopathy to either religiosity or religious affiliation. There is evidence, however, that religiosity (especially when operationalized in terms of frequency of church attendance) is negatively associated with criminal and otherwise deviant conduct (Ellis, 1985; Laird et al., 2011). Moreover, because at least some evidence points to an association between religiosity and traits associated with adequate impulse control (McCullough and Willoughby, 2009), one might anticipate that psychopathic traits, particularly those tied to risk for irresponsible and antisocial behavior, would be negatively associated with religiosity.

### Hypotheses

In light of the aforementioned literature, we undertook a preliminary examination of the correlates of psychopathic traits in everyday life using a brief internet-based survey of members of a large, non-random sample of the general population. In contrast to previous studies in non-clinical samples, almost all of which have examined the maladaptive correlates of psychopathy, we focused in part on adaptive correlates given recent suggestions that some features of psychopathy are linked to socially successful outcomes (Dutton, 2012a; Lilienfeld et al., 2012a; but see Miller and Lynam, 2012, for an alternative view). Specifically, we hypothesized that psychopathic personality traits, especially Fearless Dominance, would be linked to the adoption of leadership and management roles, as well as with occupations associated with high levels of physical risk. We also predicted that psychopathic traits, particularly Self-Centered Impulsivity, would be tied to the absence of religious belief; we advanced no specific hypotheses regarding the relation between psychopathic traits and specific religious beliefs (e.g., Protestantism, Catholicism, Judaism).

In addition to these confirmatory analyses, we conducted largely exploratory analyses. Specifically, we examined the relation between psychopathy and two other variables that bear important implications for everyday functioning, but that have received little or no explicit attention in the psychopathy literature: political affiliation and geographical region of residence. In the only published investigation to our knowledge of the relation between psychopathy and political affiliation, Hodson et al. (2009) found a non-significant association (*r* = −0.06) between psychopathy and conservatism in an undergraduate sample.

Moreover, in view of suggestive but inconclusive data that psychopathic trait levels are higher in North Americans than in Europeans (Wernke and Huss, 2008), we explored mean differences across psychopathic traits across these two continental regions. Finally, we explored differences in psychopathy levels across broad geographical regions within the United States (see Rentfrow et al., 2013, for data on personality differences across U.S. regions).

## Materials and methods

### Procedure

In January of 2013, *Scientific American Mind* magazine published an article entitled “Wisdom from Psychopaths” by Kevin Dutton (2012b), which was adapted from Dutton's (2012a) book “The Wisdom of Psychopaths.” At the conclusion of the article, readers were referred to an anonymous internet survey on psychopathy. Readers interested in participating were directed to an internet link; upon clicking the link, they received a password that permitted them to log onto a secure website. The survey instructions informed participants that they would complete “a brief questionnaire measure of psychopathic personality traits, used by many researchers to examine the levels of these traits in the general population.” They were further informed that the survey could not be used to diagnose psychopathy given that psychopathic personality traits are continuously distributed in the population, and that the survey would provide them only with an “approximate sense of how you score relative to other people of your gender on eight key aspects of psychopathic personality.”

Participants then responded to a series of demographic questions, followed by items assessing psychopathy. The survey was designed to be brief to maximize participation, and intended to be completed by participants in 15 min or less. At the conclusion of the survey, participants were given feedback, framed in socially desirable terms (e.g., high scorers on Coldheartedness were informed that they “don't have powerful emotional needs”) regarding their standing on the eight dimensions of the short form of the PPI-R (see “Measures”) relative to gender and age-matched individuals in the normative group collected for the PPI-R manual (Lilienfeld and Widows, 2005). The project was approved by the Institutional Review Board at the University of Oxford.

### Participants

The initial survey sample comprised 3618 participants. Two hundred and seventeen participants were excluded on the basis of excessive missing data on the Psychopathic Personality Inventory-Revised Short Form (see “Measures”). Eleven additional participants were excluded on the basis of extreme scores (viz., 9 or higher) on the Variable Response Inconsistency (VRIN) Scale (see Measures). Two participants were also excluded because they gave implausible responses (1337 and 6000, respectively) to the question concerning the number of lifetime leadership positions held (see “Demographic Information”). Hence, the final sample for the analyses reported here consisted of 3388 participants, although the precise sample sizes for specific analyses varied slightly depending on missing data.

Of these participants, 51.1% were female; 48.9% were male^1^. One percent of the sample had an 8th grade education or less; 6.7% had a 9th to 12th grade education but had not graduated from high school; 4.8% graduated high school but went no further; 31.7% attended fewer than four years of college; 31.8% received a bachelor's degree; 18.1% a master's degree, and 5.9% a doctoral degree. Clearly, this was a highly educated sample, as might be expected of readers of *Scientific American Mind* magazine. Information on age and race was not collected in this brief internet survey. Income levels, as measured by reported annual salaries in U.S. dollars, were as follows (with percentages in parentheses): $0–$10,000 (22.3%), $10,000–20,000 (9.7%), $20,000–$30,000 (10.5%), $30,000–$50,000 (17.5%), $50,000–$75,000 (16.2%), $100,000 (10.4%), $100,000–$150,000 (7.9%), $150,000–$200,000 (2.5%), and $200,000 and above (2.9%). The sample was predominantly (56.1%) non-religious (e.g., atheist, agnostic, non-believer, humanistic), perhaps consistent with findings that the rates of religiosity among scientists, who are presumably overrepresented among *Scientific American Mind* readers, are substantially less religious than those of members of the general population (Ecklund and Scheitle, 2007).

#### Measures

***Demographic information***. Participants recorded their gender, education, income, occupation, and political affiliation (on a scale ranging from 1 to 5, where 1 = very liberal to 5 = very conservative, and 6 being other) using drop-down menus. They indicated their occupation, religious affiliation, country of residence, and state of residence as open-ended string variables.

Participants were asked to indicate (a) whether they had ever held a position in management (e.g., boss or head of a company, head of a group project; yes, *n* = 2148; no = 1239) and (b) the total number of lifetime leadership positions they had held (e.g., president of a club or organization, political position). Occupational status was operationalized as low or high risk depending on the absence or presence of physical risk to self or others. This variable was therefore recoded into low risk (*n* = 2978) or high risk (*n* = 87), with the latter operationalized as a profession in one or more of the following fields: (a) police and other forms of law enforcement, (b) firefighting, (c) military, (d) dangerous sports (e.g., bobsledder). (e) emergency medicine, and (f) miscellaneous (e.g., pyrotechnician, lifeguard, miner, lumberjack, pilot, intelligence agent). Three-hundred and thirty-four responses were coded as missing, either because they were left blank or because they could not be coded into either low or high risk categories (e.g., “retired,” “unemployed,” “none,” “prefer not to say”). For exploratory purposes, occupations were also recoded into three subgroupings for which there were sufficient sample sizes for comparative analyses: (1) psychologist/other mental health professional (*n* = 68), (2) attorney/lawyer (*n* = 93), and (3) businessperson (*n* = 355). Although we could have conducted a number of other occupational comparisons, we elected to examine these three subgroupings in exploratory analyses given recent but preliminary assertions that individuals in the business and legal professions may be marked by especially high levels of psychopathic traits (Boddy, 2011; Smith and Lilienfeld, 2012).

Religion was recoded into the higher-order categories of non-religious vs. religious, and for exploratory purposes, more specific categories of Agnostic/Atheist/Non-religious (*n* = 1849), Protestant (*n* = 159), Catholic (*n* = 384), Jewish (92), and East Asian, including Hindu and Buddhist (79; the sample sizes of other religions were not sufficiently high to permit separate analyses).

Countries of participants were distributed across the (a) United States (79.9%), (b) Canada (7.8%), (c) Western Europe (5.1%), (d) Eastern Europe (1.1%), (e) Asia (1.9%), and Australia/New/Zealand/Tasmania (1.0%), Mexico (0.2%), and assorted countries in South America (0.3%) and Africa (0.3%). For analytic purposes, we focused our comparisons on the superordinate categories of North America (87.7%) vs. Europe (6.2%). For U.S. participants, states of residence were recoded into one of the following four standard census regions (see https://www.census.gov/const/regionmap.pdf): Northeast (17.5%), Midwest (15.8%), South (25.9%), and West (20.8%).

***Psychopathic Personality Inventory-Revised, Short Form (PPI-R-SF; Lilienfeld and Widows, 2005)***. The PPI-R-SF consists of 56 items, answered using 1–4 Likert-type anchors, developed to detect the core affective and interpersonal features of psychopathy; it does not contain items explicitly referencing criminal behavior, rendering it especially appropriate for community samples. Like the widely used 154-item PPI-R, the PPI-R-SF consists of eight subscales that coalesce into two higher-order dimensions. The PPI-R-SF subscales of Social Influence (formerly Social Potency), Fearlessness, and Stress Immunity load on the higher-order Fearless Dominance dimension; the subscales of Machiavellian Egocentricity, Carefree Non-planfulness, Rebellious Non-conformity (formerly Impulsive Non-conformity), and Blame Externalization load on the higher-order Self-Centered Impulsivity dimension; Coldheartedness does not load highly on either higher-order dimension. The PPI-R-SF was developed from its parent measure, the PPI-R, by selecting the 7 items from each of the 8 PPI-SF-R subscales that exhibited the highest loadings in factor analyses of both male and female samples (in the case of Social Influence, the 8th highest-loading item was selected to minimize content overlap given that two of the original 7 items were very similar in content). To screen out protocols for inconsistent, careless, or random responding, we developed a *post-hoc* VRIN scale (see Tellegen, 1988) by identifying the 7 PPI-R-SF item pairs with the highest (*r* > 0.60) correlations in the survey sample. Absolute score discrepancies (ranging from 0 to 3 per item) between responses to each item in the pair were summed across the 7 item pairs (see Lilienfeld and Andrews, 1996, for a description of the development of the PPI VRIN scale); as noted earlier, 11 outliers on this scale were omitted from the analyses.

Visser et al. (2012) reported that total scores on the PPI-SF-R correlate highly (*r* = 0.69) with total scores on a well-validated measure of psychopathy, the Self-Report Psychopathy Scale (Paulhus et al., in press; see also see also Marcus et al., 2013, for meta-analytic data supporting the convergent and discriminant validity of the similar short form of the PPI). In the PPI-R normative development sample (Lilienfeld and Widows, 2005), PPI-R-SF scores correlated *r* = 0.89 with total scores on the full PPI-R and *r* = 0.64 with total scores on the PCL-R (both *p*s < 0.001). The internal consistency (Cronbach's alpha) of the PPI-R-SF total score in our sample was 0.94; the internal consistencies of the PPI-R-SF Fearless Dominance, Self-Centered Impulsivity, and Coldheartedness scores were 0.86, 0.86, and 0.67, respectively^2^.

## Results

### Descriptive statistics

Descriptive statistics for the PPI-R-SF total score and higher-order dimensions for males, females, and the total sample are reported in Table 1. For these and all subsequent group difference comparisons, we adopt Cohen's (1988) approximate metrics for group differences of *d* = 0.2 is small (weak), *d* = 0.5 is medium, and *d* = 0.8 or larger is large. We first compared PPI-R-SF scores in our sample with short form scores extracted from the PPI-R combined normative sample of community members and college students (Lilienfeld and Widows, 2005; see Murray et al., 2012, for similar findings in undergraduates). For these comparisons, Cohen's *d*s were calculated by using the standard deviation from the normative sample. PPI-R-SF total (134.73 vs. 118.36; *d* = 1.07), Self-Centered Impulsivity (64.10 vs. 52.44; *d* = 0.97), and Coldheartedness (16.92 vs. 13.75; *d* = 0.88) scores were more elevated in the present sample, with all of these effects being large in magnitude. In contrast, scores on PPI-R-SF Fearless Dominance were broadly comparable in the two samples (53.71 vs. 52.16; *d* = 0.16), although there were again slightly higher in the present sample. These findings suggest that our survey sample is disproportionately weighted toward psychopathic features, at least those that tend to be maladaptive (see Discussion).

**Table 1:** **Means and standard deviations, and sex differences, for psychopathy variables**.

**Measure**	**Total sample**	**Males**	**Females**	***t***	***df***	***d***
PPI-R-SF Tot	134.73 (23.96)	141.76 (23.02)	127.99 (22.89)	17.45^*^	3385	0.60
PPI-R-SF FD	53.71 (10.70)	57.03 (10.36)	50.51 (10.03)	18.63^*^	3385	0.64
PPI-R-SF SCI	64.10 (13.10)	66.43 (12.78)	61.87 (13.03)	10.27^*^	3385	0.35
PPI-R-SF Cold	16.92 (4.65)	18.29 (4.68)	15.60 (4.22)	17.55^*^	3385	0.60

*PPI-SF-R, Psychopathic Personality Inventory-Revised, Short Form; Tot, Total score; FD, Fearless Dominance; SCI, Self-Centered Impulsivity; Cold, Coldheartedness*.

*For males, n = 1662, for females, n = 1736*.

t is test statistic for sex differences on each psychopathy variable; d = Cohen's d, computed using the pooled standard deviation.

* *p < 0.01*.

As is evident from Table 1, and consistent with the published literature on sex differences in psychopathy (Cale and Lilienfeld, 2002), males were significantly elevated on the PPI-R-SF total score and all three PPI-R-SF higher-order dimensions compared with females, with the effect sizes (Cohen's *d*s) of these differences ranging from small to medium (for PPI-R-SF Self-Centered Impulsivity) to medium to large for the other three psychopathy scores.

### Zero-order correlations

Table 2 presents the correlations between psychopathy scores and the continuously measured variables of (a) education, (b) income, (c) number of leadership positions held, and (d) political affiliation (from liberal to conservative, with higher scores coded as more conservative). Because of the very large sample size, we place primary emphasis on the magnitudes of the correlations rather than on statistical significance, although we also report the latter for the sake of completeness. Specifically, we adopt Cohen's (1988) approximate metrics for the effect size of correlations, whereby *r* = 0.1 is small, *r* = 0.3 is medium, and *r* = 0.5 or larger is large.

**Table 2:** **Correlations among dimensional variables**.

	**1**	**2**	**3**	**4**	**5**	**6**	**7**	**8**
1. PPI-R-SF Tot	−	0.85^*^	0.89^*^	0.71^*^	−0.06^*^	−0.23^*^	0.13^*^	0.20^*^
2. PPI-R:SF FD		−	0.54^*^	0.53^*^	0.06^*^	−0.11^*^	0.15^*^	0.16^*^
3. PPI-R:SF SCI			−	0.50^*^	−0.15^*^	−0.29^*^	0.09^*^	0.19^*^
4. PPI-R:SF COLD				−	−0.01	−0.14^*^	0.05^*^	0.20^*^
5. Income					−	0.40^*^	0.07^*^	−0.07^*^
6. Education						−	−0.01	−0.23^*^
7. Leadership							−	0.02
8. Conservatism								−

PPI-R-SF, Psychopathic Personality Inventory-Revised, Short Form; Tot, Total score; FD, Fearless Dominance; SCI, Self-Centered Impulsivity; Cold, Coldheartedness; Education, Educational Level; Leadership, Number of Lifetime Leadership Positions; Conservatism, Degree of Political Conservatism.

* *p < 0.01; N = 3387*.

PPI-R-SF total scores and all three PPI-R-SF higher-order dimensions were negatively and modestly correlated with educational level, with the association most marked for Self-Centered Impulsivity. PPI-R-SF total scores were negatively correlated with income level, although this association did not even attain the level of a weak effect size. In contrast to the correlations for educational level, PPI-R-SF Fearless Dominance and Self-Centered Impulsivity were correlated with income in opposing directions, with the former being weakly positively associated and the latter weakly negatively associated.

PPI-R-SF total scores were positively, albeit weakly, associated with the number of leadership positions held. Although all three PPI-R-SF higher-order dimensions were associated with number of leadership positions, this association was, as predicted, significantly more marked for Fearless Dominance than for other PPI-R-SF dimensions. Tests of the significance of the difference between dependent correlations (Steiger, 1980) revealed that the PPI-R-SF Fearless Dominance correlation with number of leadership positions significantly exceeded both those of PPI-R-SF Self-Centered Impulsivity: *z*_(3385)_ = 4.05, *p* < 0.001; and PPI-R-SF Coldheartedness: *z*_(3385)_ = 6.35, *p* < 0.001 (controlling statistically for PPI-R-SF Self-Centered Impulsivity scores using partial correlation left the association between PPI-R-SF Fearless Dominance and leadership positions intact; the converse analysis controlling for PPI-R-SF Fearless Dominance scores did not substantially change the association between PPI-R-SF Self-Centered Impulsivity and leadership positions).

PPI-R-SF total, Fearless Dominance, Self-Centered Impulsivity, and Coldheartedness scores were weakly but positively correlated with political conservatism. Subsidiary partial correlation analyses revealed that these associations remained essentially unchanged after controlling statistically for income and educational level (controlling statistically for PPI-R-SF Self-Centered Impulsivity scores similarly left the association between PPI-R-SF Fearless Dominance and leadership positions intact; the converse analysis controlling for PPI-R-SF Fearless Dominance scores again did not substantially change the association between PPI-R-SF Self-Centered Impulsivity and leadership positions). Given that political liberalism and conservatism often carry different meanings in the United States as opposed to some European countries (Collins, 1993), we re-conducted the zero-order correlational analyses within the United States alone; the results remained virtually identical, with the changes in *r*s across for four PPI-R-SF variables ranging from 0.00 to 0.01.

### Group differences

Table 3 displays the results of *t*-tests of group differences for which we had explicit hypotheses, namely, for management vs. non-management roles, high occupational risk vs. low occupational risk, and religiously affiliated vs. non-religiously affiliated (the results of exploratory analyses are further described in the text). Cohen's *d*s for these and subsequent group comparisons were calculated using the pooled standard deviation across groups.

**Table 3:** **Group comparisons for managerial status, high-risk occupational status, and religious status**.

**Measure**	**Managerial position (Yes)**	**Managerial position (No)**	***t***	***df***	***d***
PPI-R-SF Tot	136.54 (23.92)	131.58 (23.70)	5.83^*^	3385	0.21
PPI-R-SF FD	55.10 (10.39)	51.30 (10.39)	10.11^*^	3385	0.36
PPI-R-SF SCI	64.43 (13.04)	63.53 (13.20)	1.92	3385	0.07
PPI-R-SF Cold	17.00 (4.56)	16.75 (4.78)	1.55	3385	0.05
**Measure**	**Occupational risk (High)**	**Occupational risk (Low)**	***t***	***Df***	***d***
PPI-R-SF Tot	145.66 (22.12)	133.95 (23.72)	4.55^*^	3053	0.51
PPI-R-SF FD	59.31 (9.49)	53.57 (10.65)	4.96^*^	3053	0.57
PPI-R-SF SCI	67.45 (12.60)	63.62 (12.90)	2.73^*^	3053	0.30
PPI-R-SF Cold	18.90 (4.64)	16.75 (4.56)	4.32^*^	3053	0.47
**Measure**	**Non-religious**	**Religious**	***t***	***Df***	***d***
PPI-R-SF Tot	136.01 (24.54)	132.76 (23.01)	3.89^*^	3281	0.14
PPI-R-SF FD	53.72 (10.90)	53.47 (10.47)	0.65	3281	0.02
PPI-R-SF SCI	64.86 (13.21)	63.04 (12.81)	3.99^*^	3281	0.14
PPI-R-SF Cold	17.43 (4.67)	16.25 (4.49)	7.39^*^	3281	0.26

*PPI-SF-R, Psychopathic Personality Inventory-Revised, Short Form; Tot, Total score; FD, Fearless Dominance; SCI, Self-Centered Impulsivity; Cold, Coldheartedness*.

*For managerial position (yes), n = 2148, for managerial position (no), n = 1239; for occupational risk (high), n = 87, for occupational risk (low), n = 2968; for non-religious, n = 1773, for religious, n = 1510*.

t is test statistic for group differences on each psychopathy variable; d = Cohen's d, computed using the pooled standard deviation.

* *p < 0.01*.

First, we compared psychopathy scores among individuals who had reported a history of being in a managerial role and individuals who had not. Individuals with a managerial history obtained higher PPI-R-SF total and Fearless Dominance scores than those without such a history, with the effect sizes being small and small to medium, respectively. Analyses of covariance (not reported here) controlling statistically for educational level yielded no substantive changes in the pattern of results (these analyses are available from the first author on request). As can also be seen in Table 3, individuals in high-risk occupations obtained significantly higher scores on all four PPI-R-SF variables. The effect sizes were medium for the PPI-R-SF Total, Fearless Dominance, and Coldheartedness scores, and small to medium for PPI-R-SF Self-Centered Impulsivity.

An analysis of variance comparing the three major occupational types we examined (psychologist/other mental health professional, attorney/lawyer, and businessperson) were statistically significant for PPI-R-SF total scores [*F*_(2, 513)_ = 3.99, *p* = 0.019] and PPI-R-SF Self-Centered Impulsivity scores [*F*_(2, 513)_ = 4.16, *p* = 0.016], but not for the other two PPI-R-SF variables. Tukey Honestly Significant Difference (HSD) *post-hoc* tests revealed that the source of the omnibus difference for the PPI-R-SF total score was the significantly higher score of businesspersons [*M* = 135.23 (SD = 22.50)] compared with psychologists/mental health professionals [*M* = 127.03 (SD = 21.59)], *p* = 0.015. This difference was small to medium in magnitude (*d* = 0.37). Turkey HSD tests again revealed that the source of the omnibus difference for PPI-R-SF Self-Centered Impulsivity scores was the significantly higher score of businesspersons [*M* = 63.34 (SD = 12.64)] compared with psychologists/mental health professionals [*M* = 58.75 (SD = 11.92)], *p* = 0.013. This difference was also small to medium in magnitude, and was identical to the previous effect size (*d* = 0.37).

We next examined differences in psychopathy scores across religious groups. As displayed in Table 3, independent sample *t*-tests revealed that compared with religious individuals, non-religious individuals were significantly more elevated on PPI-R-SF total, Self-Centered Impulsivity, and Coldheartedness scores; differences for PPI-R-SF Fearless Dominance were not statistically significant. The effect sizes for the non-religious-religious differences for PPI-R-SF Total, Self-Centered Impulsivity, and Coldheartedness scores were all weak in magnitude, with only the lattermost difference reaching Cohen's criteria for a small effect size (subsidiary analyses of covariance controlling for educational levels left these differences intact). An analysis of variance comparing psychopathy levels across the five groups with sufficient sample sizes (Agnostic/Atheist/Non-religious, Protestant, Catholic, Jewish, and East Asian) was statistically significant for PPI-R-SF total [*F*_(4, 2552)_ = 6.91, *p* < 0.001], PPI-R-SF Fearless Dominance [*F*_(2, 2552)_ = 2.61, *p* = 0.034], PPI-R-SF Self-Centered Impulsivity [*F*_(2, 2552)_ = 0.8.25, *p* < 0.001], and PPI-R-SF Coldheartedness [*F*_(2,2552)_ = 6.92, *p* < 0.001] scores. Tukey HSD follow-up tests revealed that for PPI-R-SF total, Self-Centered Impulsivity, and Coldheartedness scores, the source of this difference was the same, namely, the higher scores of non-religious individuals compared with Protestants (for PPI-SF total, Self-Centered Impulsivity, and Coldheartedness, *p*s < 0.001 for all three variables) and Jews (*p*s = 0.002, 0.004, and 0.029, respectively). For PPI-R-SF Fearless Dominance scores, the source of the difference was the significantly higher scores of Protestants than Jews (*p* = 0.049).

Finally, we compared the psychopathy scores of participants across geographical regions, first focusing on North America vs. Europe. Independent sample *t*-tests revealed that European participants were significantly more elevated than North American participants on PPI-R-SF total [*t*_(3175)_ = 3.83, *p* < 0.001; *d* = 0.27], PPI-R-SF Self-Centered Impulsivity [*t*_(3175)_ = 4.40, *p* < 0.001, *d* = 0.33], and PPI-R-SF Coldheartedness [*t*_(3175)_ = 3.40, *p* = 0.001, *d* = 0.22] scores, with effect sizes in the small to medium range. The differences for PPI-R-SF Fearless Dominance were in the same direction but fell short of significance [*t*_(3175)_ = 1.74, *p* = 0.08, *d* = 0.12] (subsidiary analyses excluding Eastern Europeans and focused on Western Europeans only yielded very similar results for all psychopathy variables). An analysis of variance examining psychopathy scores across the four major U.S. geographical census tract regions (Northeast, Midwest, South, West) yielded no significant differences for any of the four PPI-R-SF variables (all *F*s ≤ 1.62, all *p*s > 18).

## Discussion

The extent to which personality traits are correlated with important domains of everyday functioning, including occupational choice, work performance, political affiliation, and religiosity, has become an increasing focus of research in recent years (see Ozer and Benet-Martinez, 2006 and Roberts et al., 2007, for reviews). Much of this literature suggests that, despite initial skepticism (Mischel, 1968), many personality traits bear statistically and practically significant relations with important real-world behavioral outcomes. Surprisingly, there has been scant investigation of this question with respect to psychopathy, a clinically important condition marked by extreme levels of several personality trait dimensions (Patrick et al., 2009). As an initial step toward filling this gap, we undertook a preliminary investigation of this issue by using an internet-based survey of a large (*N* = 3388) general population sample. In contrast to most previous studies of psychopathy in the community, we focused largely on variables relevant to adaptive functioning, especially leadership.

Our analyses, although limited to a small number of items given the marked time constraints of our survey methodology, yielded several novel findings. Most of our statistical associations were, however, small or at best medium in magnitude. Nevertheless, even small effect sizes can bear important real-world implications at the population level. For example, in large sample studies, the correlation between combat exposure and risk for posttraumatic stress disorder is *r* = 0.11, and the correlation between lead exposure and childhood IQ is *r* = −0.12 (Meyer et al., 2001). Both of these well-accepted statistical associations, although small by Cohen's (1988) standards, bear enormous mental and public health implications at the level of the broader population.

Our study provides the first published evidence that psychopathic traits, especially Fearless Dominance, which is similar to boldness (Patrick et al., 2009), are tied to an elevated probability of occupying leadership and management positions, Nevertheless, the magnitudes of these relations were modest. Corroborating the conjectures of some previous authors (e.g., Lykken, 1995; Dutton, 2012a), we found that psychopathic propensities are associated with holding high-risk occupations, such as police work and firefighting (see also Falkenbach and Tsoukalas, 2011). This finding, which was medium in size for Fearless Dominance, is consistent with previous theoretical and empirical work on “niche picking,” which suggests that individuals with certain personality traits may tend to select occupations and avocations that afford behavioral expressions of their dispositions (Ickes et al., 1997). Nevertheless, because our methodology was cross-sectional, we cannot exclude the hypothesis that high-risk occupations, such as law enforcement, themselves increase certain traits relevant to psychopathy, such as fearlessness, via repeated exposure to fear-provoking situations. In addition, alternative explanations for this finding are possible (see Roberts et al., 2003, for a broader discussion). For example, law enforcement agencies may tend to select individuals with elevated levels of fearlessness or certain other psychopathic traits, or individuals with low levels of these traits may be less likely than individuals with high levels to remain in dangerous professions. Further research using longitudinal designs should help to clarify the temporal course, and perhaps causal directionality, of the relation between psychopathic traits and high-risk occupations.

Our study also provides the first systematic evidence that psychopathic traits bear implications for political affiliation (cf., Hodson et al., 2009), although the magnitudes of these statistical effects were again modest. The finding that psychopathic traits are slightly associated with political conservatism was not predicted, and may appear to conflict with findings that (a) conservatives base their morality on a broader set of foundations (e.g., fairness, care, loyalty, respect, sanctity) than do liberals (Graham et al., 2009) and (b) psychopathic individuals base their morality on weaker foundations, especially harm and fairness, than do non-psychopathic individuals (Glenn et al., 2009). At the same time, our findings are open to multiple interpretations. In particular, it is unclear whether our findings are attributable to cultural (social) conservatism, economic conservatism, or both. This ambiguity is important given that cultural and economic conservatism tend to be only weakly correlated and to display differing personality correlates (Crowson, 2009). It is also possible that our measure of conservatism served as a proxy for social dominance orientation, viz., a preference for social group inequality (Pratto et al., 1994), a trait that is moderately correlated with, but separable from, conservatism. Because social dominance orientation is moderately associated with psychopathy (Hodson et al., 2009), this possibility should be investigated in future research.

Our findings also bear intriguing implications for the response penetration of psychopathy into the religious domain. We found that religious non-believers reported higher levels of psychopathic traits, namely Self-Centered Impulsivity and Coldheartedness, than do religious believers, although only the magnitude for Coldheartedness reached Cohen's cut-off for a small effect size. Although this is the first reported finding of differences in psychopathic trait levels between religious believers and non-believers, it may dovetail with findings of higher levels of disinhibition and antisocial behavior among the latter group (Rohrbaugh and Jessor, 1975; Free, 1992). It is unclear, however, whether this association is causal (Cochran et al., 1994). In exploratory analyses, we found that non-believers received higher scores on the maladaptive features of psychopathy, namely Self-Centered Impulsivity and Coldheartedness, compared with Jews and Protestants. Interestingly, in a narrative review of over 50 studies, Ellis (1985) reported that Jews tend to have somewhat lower crime rates than Christians as a whole, and that among Christians, Protestants tend to have somewhat lower crime rates than Jews. Nevertheless, because the overlap between psychopathy and criminality is only modest (Hart and Hare, 1997), the extent to which these conclusions bear on the present findings requires clarification.

Finally, we also examined cross-national differences in psychopathy. We found that Europeans, including Western Europeans, obtained significantly higher scores than did North Americans on measures of the maladaptive features of psychopathy, with effect sizes in the small to medium range. These results seemingly run counter to prior reports of lower psychopathy scores among European than among North American prisoners (e.g., Cooke and Michie, 1999), and may buttress contentions that previous reports of differences between North Americans and Europeans are attributable to selection biases in the criminal justice system (Wernke and Huss, 2008). Nevertheless, we cannot exclude the possibility that our findings are themselves due to an undetected differential selection artifact (see Campbell and Stanley, 1963), whereby North Americans and Europeans who elected to complete our internet survey differed on one or more unmeasured variables (e.g., criminal history) that are themselves correlated with psychopathy.

Our study was marked by a number of strengths, particularly its large sample size and use of a well-validated measure of psychopathy. At the same time, our study was characterized by several limitations, several of which offer fruitful directions for future research. Because our study was cross-sectional, we are necessarily precluded from drawing longitudinal, let alone causal, conclusions. In addition, owing largely to the need to keep our survey exceedingly brief, we collected data on only one measure of psychopathy; we did not collect complete demographic information; we relied on single-item indicators of leadership and management positions; and we did not collect data on other personality disorders or on normal range personality traits (e.g., dimensions of the widely used five-factor model). As a consequence of the lattermost limitation, the extent to which our findings are specific to psychopathy *per se* as opposed to other personality disorders, such as narcissistic personality disorder, are unclear. For example, narcissistic personality traits have been linked to leadership emergence and perhaps success (Brunell et al., 2008; Watts et al., 2013) and hence would be worth examining in conjunction with psychopathy in future survey research.

Given our survey methodology, we were limited to self-reports of psychopathic traits and demographic/life history variables. Hence, one potential threat to the validity of our results is response bias. In particular, psychopathic individuals' well-known propensity toward prevarication (Cleckley, 1941; Lilienfeld and Fowler, 2006) may render our findings vulnerable to the possibility that the association between psychopathic traits and occupational variables (e.g., leadership positions) is attributable to positive impression management. Although this possibility cannot be excluded with certainty, meta-analytic findings suggest that scores on psychopathy questionnaires tend to be *negatively* associated with measures positive impression management (Ray et al., 2013). These results suggest that individuals with psychopathic traits are more than willing to admit to socially undesirable attributes, at least in research settings.

In addition, the generalizability of our findings may be limited by selection bias; indeed, comparisons of our mean scores with those of previous samples pointed to elevated levels of psychopathic traits, namely Self-Centered Impulsivity and Coldheartedness, which are linked to maladaptive outcomes. This likely selection effect may have resulted from the fact that the *Scientific American Mind* article that preceded this survey (Dutton, 2012b) was explicitly about psychopathy. Moreover, because readers who were sufficiently motivated to complete the survey were presumably interested in obtaining feedback regarding their psychopathy scores, it is not unreasonable to surmise that they may have been disproportionately psychopathic themselves. Although these selection factors appear to have fortuitously resulted in a sample enriched with high levels of psychopathic traits, further research will be needed to determine whether our findings generalize to community samples with lower levels of these traits. In this respect, our design—and its potential external validity limitations—may be analogous to other designs that have recruited individuals at elevated risk for psychopathy, such as newspaper advertisement methodologies targeting individuals with pronounced psychopathic traits (e.g., Widom, 1977; Miller et al., 2014). Nevertheless, because the aim of our study was to examine the interrelations between psychopathy and everyday life variables rather than to estimate the mean levels of psychopathy in the general population, this selection bias seems unlikely (barring a statistical interaction between selection bias and our measured variables, which have no reason to anticipate; Cook and Campbell, 1979), to threaten the validity of our correlational and group difference findings.

In further survey research on the general population, it will be essential to extend our preliminary results to additional variables of real-world importance, such as interpersonal outcomes (e.g., marital and divorce history, relationship fidelity), employment history, academic performance, criminal behavior, substance abuse, and everyday heroism (see Smith et al., 2013). The extent to which psychopathic traits are linked to adaptive as well as maladaptive interpersonal outcomes remains a topic of lively scholarly debate (e.g., Miller and Lynam, 2012; Lilienfeld et al., 2012a; Patrick et al., 2013).

Although our results do not resolve this controversy, they are consistent with the hypothesis at least some psychopathic traits, especially those relevant to social and physical boldness, are linked to adaptive attributes in everyday life, including leadership positions, management positions, and high-risk occupations. Future survey work in community samples should strive to shed additional light on this contentious question, as well as on the theoretically and pragmatically significant issue of psychopathy's relations to everyday functioning.

### Conflict of interest statement

The authors declare that the research was conducted in the absence of any commercial or financial relationships that could be construed as a potential conflict of interest.

## Footnotes

^1^Moderated multiple regression analyses (using gender as a categorical moderator) revealed no substantial evidence for differences in the relations between PPI-R-SF total scores and education, income, number of leadership positions, or political affiliation between males and females (all *R*^2^ changes for the interaction term were 0.002 or lower).

^2^Following the PPI-R-SF, participants received the 4 items assessing narcissism from the “Dirty Dozen” measure of the dark triad (psychopathy, narcissism, Machiavellianism) of personality (Jonason and Webster, 2010). This scale correlated r = 0.40, 0.53, and 0.33, with PPI-R-SF Fearless Dominance, Self-Centered Impulsivity, and Coldheartedness, Nevertheless, because the internal consistency of this scale was inadequate (Cronbach's alpha = 0.48), it was not analyzed further.
